# Supplementary material for: Chemical Diversity of Metal Sulfide Minerals and Its Implications for the Origin of Life
Source: Life (Basel). 2018 Oct 10;8(4):46. doi: 10.3390/life8040046 (PMC6316247; doi:10.3390/life8040046)
Supplement: Supplementary file 1 [file life-08-00046-s001.pdf]

Chemical Diversity of Metal Sulfide Minerals and its Implications for Origin of Life

Yamei Li [1]*, Norio Kitadai [1] and Ryuhei Nakamura [1,2]*

[1] Earth-Life Science Institute, Tokyo Institute of Technology, 2-12-1 Ookayama, Meguro-ku, Tokyo 152-8550, Japan;
[2] Biofunctional Catalyst Research Team, RIKEN Center for Sustainable Resource Science, 2-1 Hirosawa, Wako, Saitama 351-0198, Japan;
* Correspondence: yamei.li@elsi.jp; ryuhei.nakamura@riken.jp; Tel.: +81-3-5734-3414

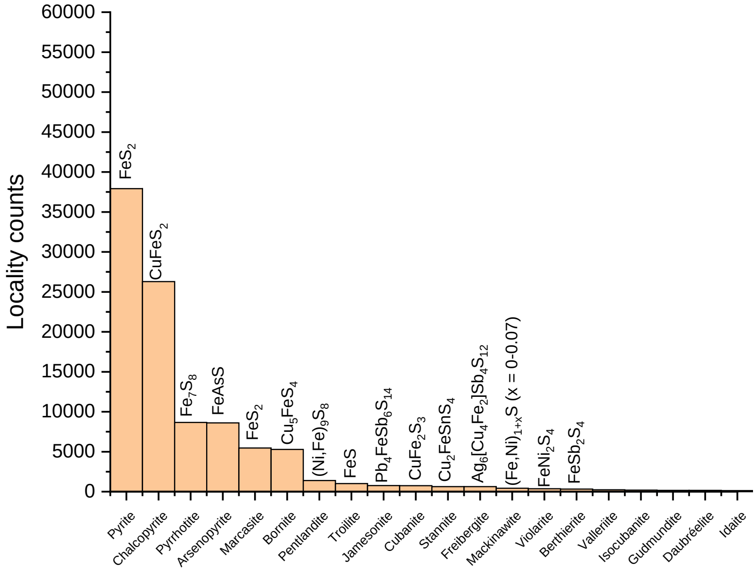

Figure S1. Species-locality distribution of Fe-containing sulfides (top 20).

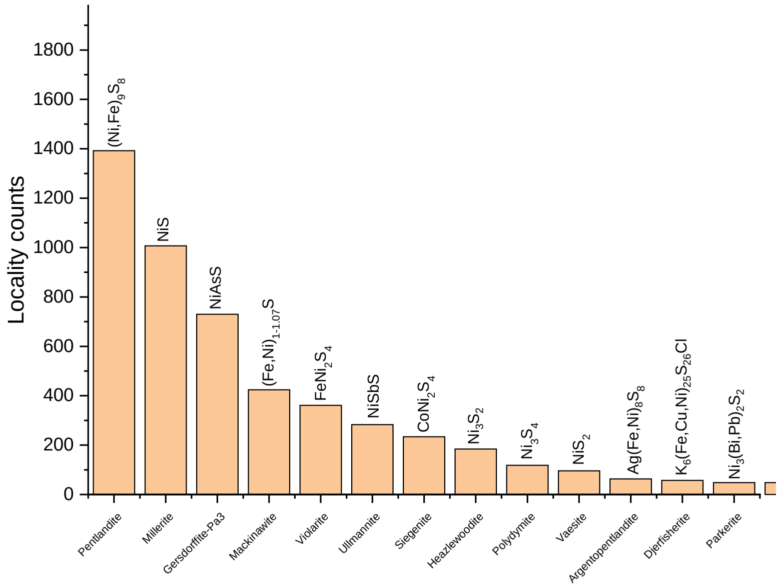

Figure S2. Species-locality distribution of Ni-containing sulfides (top 12).

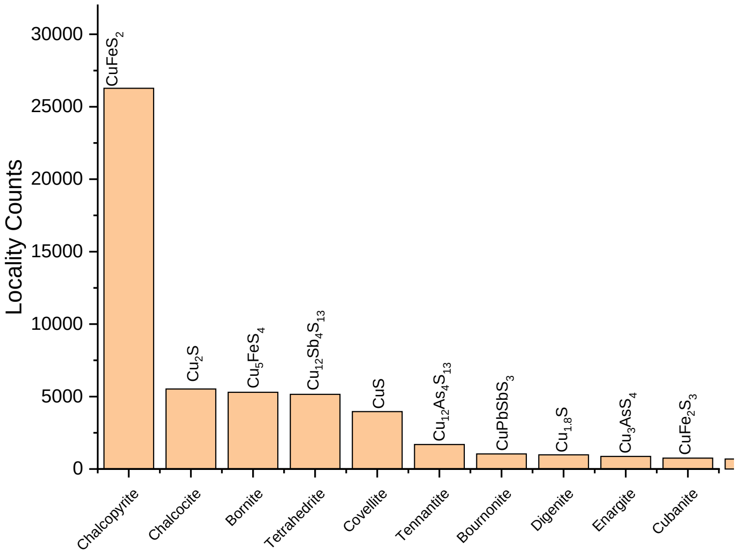

Figure S3. Species-locality distribution of Cu-containing sulfides (top 10).

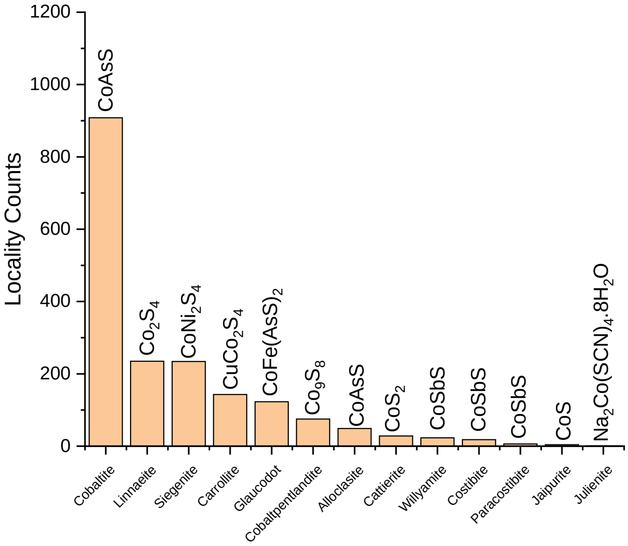

Figure S4. Species-locality distribution of Co-containing sulfides.

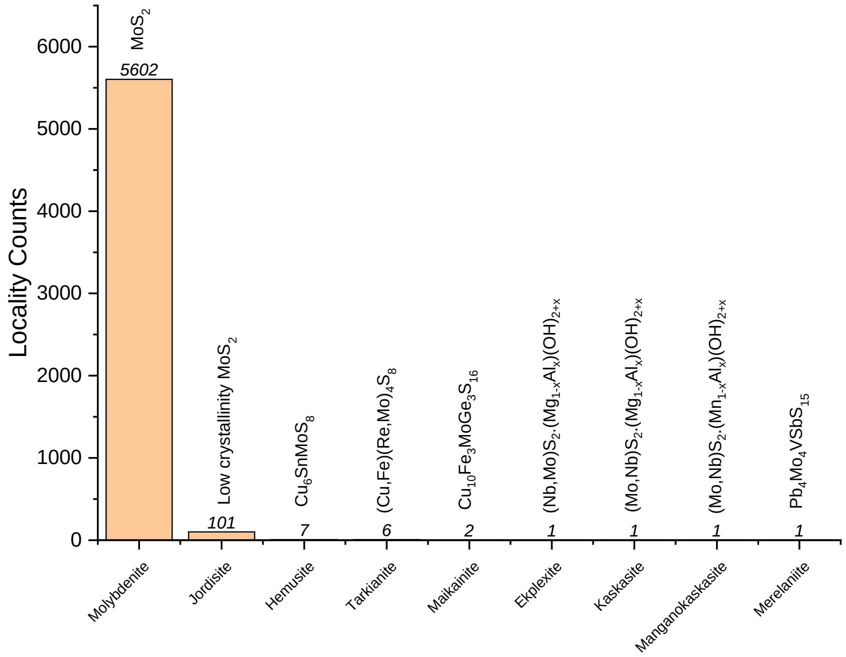

Figure S5. Species-locality distribution of Mo-containing sulfides.

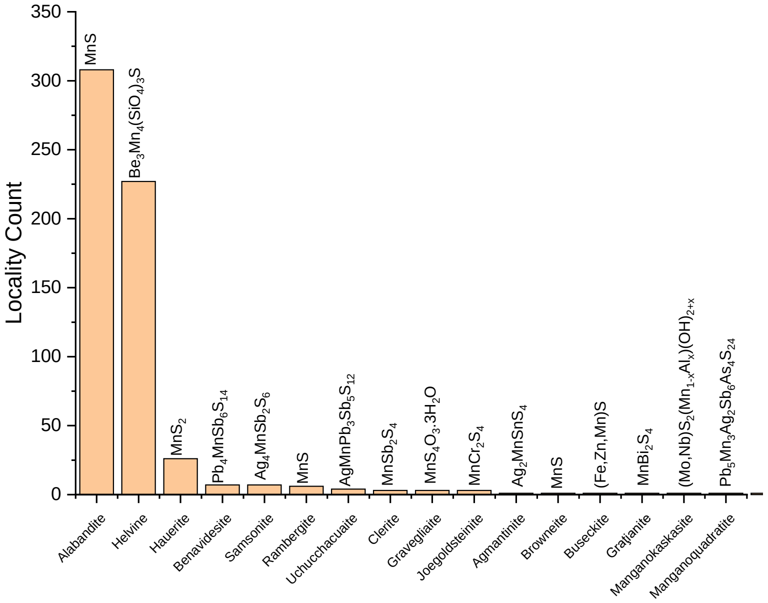

Figure S6. Species-locality distribution of Mn-containing sulfides.

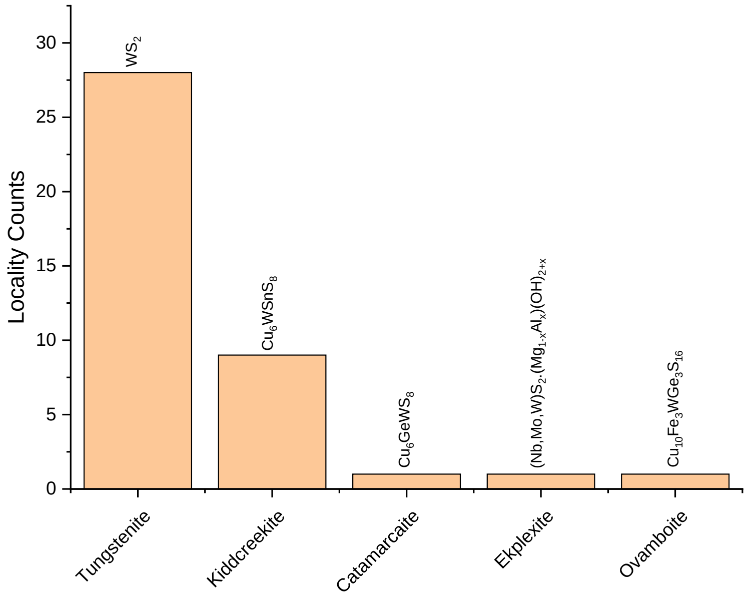

Figure S7. Species-locality distribution of W-containing sulfides.

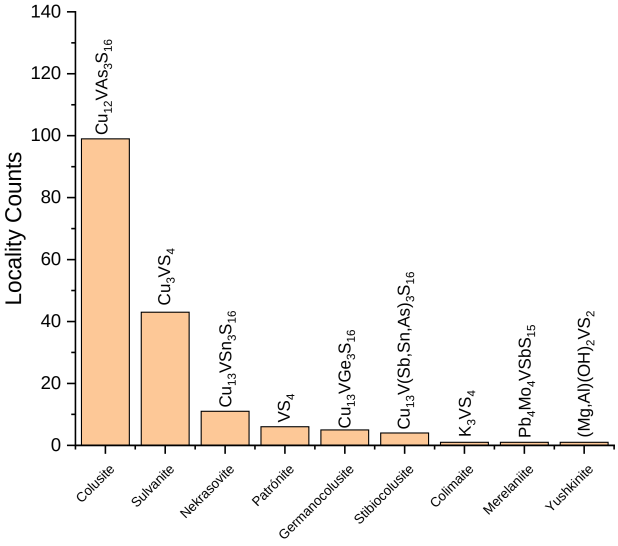

Figure S8. Species-locality distribution of V-containing sulfides.

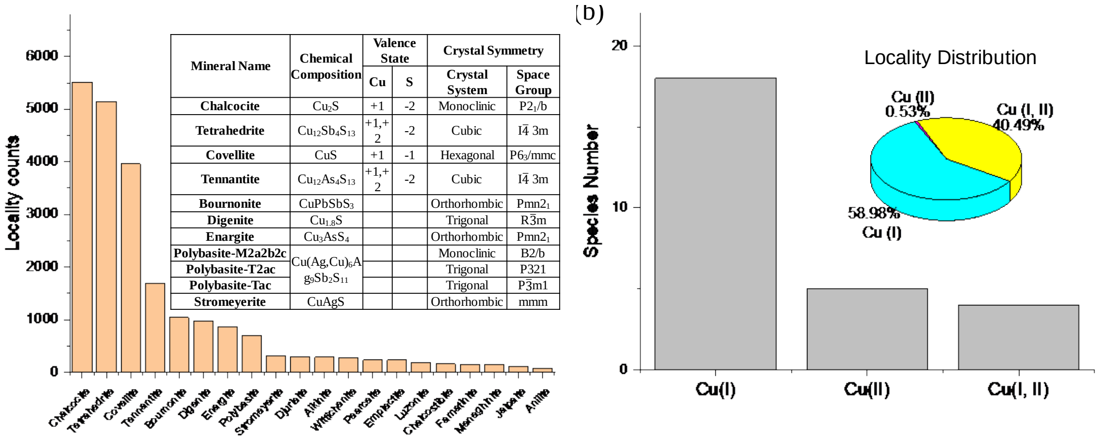

Mineral Name	Chemical Composition	Valence State		Crystal Symmetry	
		Cu	S	Crystal System	Space Group
**Chalcocite**	Cu_2S	+1	-2	Monoclinic	$P2_1/b$
**Tetrahedrite**	$Cu_{12}Sb_4S_{13}$	+1,+2	-2	Cubic	$I\bar{4}\,3m$
**Covellite**	CuS	+1	-1	Hexagonal	$P6_3/mmc$
**Tennantite**	$Cu_{12}As_4S_{13}$	+1,+2	-2	Cubic	$I\bar{4}\,3m$
**Bournonite**	$CuPbSbS_3$			Orthorhombic	$Pmn2_1$
**Digenite**	$Cu_{1.8}S$			Trigonal	$R\bar{3}m$
**Enargite**	Cu_3AsS_4			Orthorhombic	$Pmn2_1$
**Polybasite-M2a2b2c**	$Cu(Ag,Cu)_6Ag_9Sb_2S_{11}$			Monoclinic	B2/b
**Polybasite-T2ac**				Trigonal	P321
**Polybasite-Tac**				Trigonal	$P\bar{3}m1$
**Stromeyerite**	CuAgS			Orthorhombic	mmm

Figure S9. (a) Locality-mineral distribution of Cu mono-metal sulfide species and chemical diversity with regards to chemical composition, Cu/S valence states and crystal symmetry; (b) plots of the distribution of species type and locality counts for species containing Cu(I), Cu(II) and Cu(I,II) valence states.

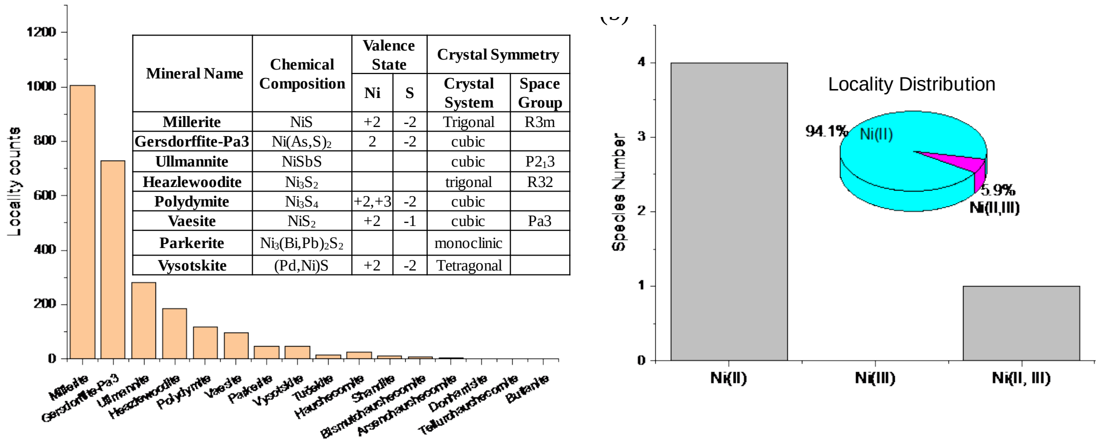

Mineral Name	Chemical Composition	Valence State		Crystal Symmetry	
		Ni	S	Crystal System	Space Group
**Millerite**	NiS	+2	-2	Trigonal	R3m
**Gersdorffite-Pa3**	$Ni(As,S)_2$	2	-2	cubic	
**Ullmannite**	NiSbS			cubic	$P2_13$
**Heazlewoodite**	Ni_3S_2			trigonal	R32
**Polydymite**	Ni_3S_4	+2,+3	-2	cubic	
**Vaesite**	NiS_2	+2	-1	cubic	Pa3
**Parkerite**	$Ni_3(Bi,Pb)_2S_2$			monoclinic	
**Vysotskite**	(Pd,Ni)S	+2	-2	Tetragonal	

Figure S10. (a) Locality-mineral distribution of Ni mono-metal sulfide species and chemical diversity with regards to chemical composition, Ni/S valence states and crystal symmetry; (b) plots of the distribution of species type and locality counts for species containing Ni(II), Ni(III) and Ni(II,III) valence states.

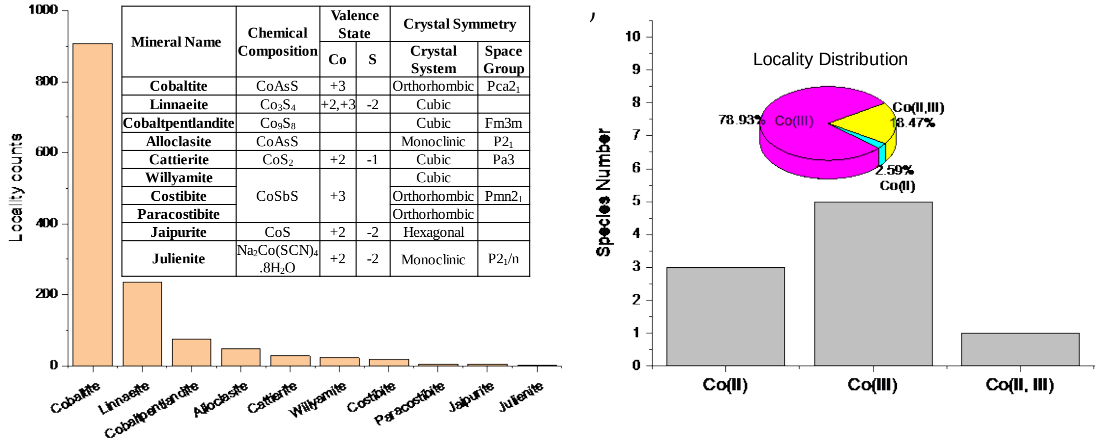

Figure S11. (a) Locality-mineral distribution of Co mono-metal sulfide species and chemical diversity with regards to chemical composition, Co/S valence states and crystal symmetry; (b) plots of the distribution of species type and locality counts for species containing Co(II), Co(III) and Co(II,III) valence states.

Table S1. Chemical properties of Fe-Cu and Fe-Ni binary metal sulfide minerals.

Fe, Cu							
**Mineral Name**	**Locality Counts**	**Chemical Composition**	**Valence State**			**Crystal Symmetry**	
			**Cu**	**Fe**	**S**	**Crystal System**	**Space Group**
**Chalcopyrite**	26279	$CuFeS_2$	+1	+3	-2	Tetragonal	I42d
**Bornite**	5293	Cu_5FeS_4	+1	+3	-2	Orthorhombic	Pbca
**Cubanite**	748	$CuFe_2S_3$	+1	+2,+3	-2	Orthorhombic	2/m2/m2/m
**Stannite**	632	$Cu_2FeSn_4S_4$	+1	+2	-2	Tetragonal	I42m
**Freibergite**	630	$Ag_6Cu_4Fe_2Sb_4S_{12}$	+1	+2	-2	Tetragonal	I43m
**Valleriite**	229	$2[(Fe,Cu)S]\cdot1.53[(Mg,Al)(OH)_2]$	+2	+2	-2	Hexagonal	
**Isocubanite**	184	$CuFe_2S_3$	+1	+2,+3	-2	Cubic	Fm3m
**Idaite**	133	Cu_3FeS_4	+2	+2	-2	Hexagonal	

Fe, Ni							
**Mineral Name**	**Locality Counts**	**Chemical Composition**	**Valence State**			**Crystal Symmetry**	
			**Fe**	**Ni**	**S**	**Crystal System**	**Space Group**
**Pentlandite**	1392	$(Ni,Fe)_9S_8$				Cubic	Fm3m
**Mackinawite**	424	$(Fe,Ni)_{1+x}S$ (x = 0-0.07)	+2	+2	-2	Tetragonal	P4/nmm
**Violarite**	361	$FeNi_2S_4$	+2	+3	-2	Cubic	
**Smythite**	71	$(Fe,Ni)_{3+x}S_4$ (x ≈ 0-0.3)				Trigonal	$R\bar{3}m$
**Argentopentlandite**	63	$Ag(Fe,Ni)_8S_8$				Cubic	Fm3m
**Godlevskite**	32	$(Ni,Fe)_9S_8$				Orthorhombic	C222

Table S2. Sulfide minerals with ternary metal compositions.

Mineral Name	Locality Counts	Chemical Composition
**Djerfisherite**	57	$K_6(Fe,Cu,Ni)_{25}S_{26}Cl$
**Sugakiite**	3	$Cu(Fe,Ni)_8S_8$
**Kharaelakhite**	2	$(Cu,Pt,Pb,Fe,Ni)_9S_8$
**Owensite**	2	$(Ba,Pb^{2+})_6(Cu^{1+},Fe^{2+},Ni^{2+})_{25}S^{2-}_{27}$
**Samaniite**	2	$Cu_2Fe_5Ni_2S_8$
**Ferhodsite**	1	$(Fe,Rh,Ni,Ir,Cu,Pt)_9S_8$
**Zoharite**	1	$(Ba,K)_6(Fe,Cu,Ni)_{25}S_{27}$
**Tarkianite**	6	$(Cu,Fe)(Re,Mo)_4S_8$
**Maikainite**	2	$Cu^{1+}_{10}Fe^{2+}_3Mo^{4+}Ge^{4+}_3S^{2-}_{16}$
**Ovamboite**	1	$Cu^{1+}_{10}Fe^{2+}_3W^{4+}Ge^{4+}_3S^{2-}_{16}$

Table S3. X-ray amorphous mineral species in the RRUFF database.

Mineral Name	Chemical Composition
**Delvauxite**	$CaFe^{3+}_4(P^{5+}O_4)_2(OH)_8$. 4-5$H_2O$
**Diadochite**	$Fe^{3+}_2(PO_4)(SO_4)(OH)$. 6$H_2O$
**Ekanite**	$Ca_2ThSi_8O_{20}$
**Evansite**	$Al_3PO_4(OH)_6$. 8H_2O
**Georgeite**	$Cu^{2+}_2CO_3(OH)_2$
**Ice**	H_2O
**Jordisite**	$Mo^{4+}S^{2-}_2$
**Zaratite**	$Ni^{2+}_3C^{4+}O_3(OH)_4$. 4H_2O
